# Outcomes of Inflammatory Bowel Disease in Hospitalized Patients With Generalized Anxiety Disorder

**DOI:** 10.7759/cureus.27656

**Published:** 2022-08-03

**Authors:** Alexander J Kaye, Shivani J Patel, Sarah R Meyers, Sushil Ahlawat

**Affiliations:** 1 Internal Medicine, Rutgers University New Jersey Medical School, Newark, USA; 2 Psychiatry, Rutgers University Robert Wood Johnson Medical School, Piscataway, USA; 3 Gastroenterology and Hepatology, Rutgers University New Jersey Medical School, Newark, USA

**Keywords:** inpatient mortality, generalized anxiety disorder, inflammatory bowel disease, intestinal perforation, intestinal abscess, acute respiratory failure, acute hepatic failure, sepsis

## Abstract

Background

The development of inflammatory bowel disease (IBD), which encompasses ulcerative colitis and Crohn’s disease, is multifactorial. Stress from anxiety is a risk factor for IBD. Generalized anxiety disorder (GAD) is twice as likely in IBD patients. This study explores the outcomes of patients hospitalized for IBD with comorbid GAD.

Methods

A retrospective analysis utilizing the 2014 USA National Inpatient Sample database was performed to assess the outcomes of hospitalized IBD patients with and without GAD. The outcomes analyzed were sepsis, acute hepatic failure, hypotension/shock, acute respiratory failure, acute deep vein thrombosis, acute renal failure, intestinal obstruction, myocardial infarction, ileus, inpatient mortality, colectomy, intestinal abscess, intestinal perforation, and megacolon. A multivariate logistic regression analysis was employed to explore whether GAD is a risk factor for these outcomes.

Results

Among 28,173 IBD hospitalized patients in the study, GAD was a comorbid diagnosis in 3,400 of those patients. IBD patients with coexisting GAD were found to be at increased risk for acute hepatic failure (adjusted odds ratio (aOR) 1.80, p = 0.006), sepsis (aOR 1.33, p < 0.001), acute respiratory failure (aOR 1.24, p = 0.018), inpatient mortality (aOR 1.87, p < 0.001), intestinal abscess (aOR 2.35, p = 0.013), and intestinal perforation (aOR 1.44, p = 0.019). The aORs for the remaining outcomes were not statistically significant.

Conclusions

In hospitalized IBD patients, GAD is a risk factor for sepsis, acute hepatic failure, acute respiratory failure, intestinal abscess, intestinal perforation, and inpatient mortality. IBD and GAD are becoming increasingly common, which will likely lead to a larger number of complications among inpatients with these comorbidities.

## Introduction

Inflammatory bowel disease (IBD) is a gastrointestinal pathology encompassing both ulcerative colitis (UC) and Crohn’s disease (CD) [1]. IBD is a chronic condition with increased morbidity and disability [2]. During the 2004 year, approximately one million people in the United States of America (USA) and 2.5 million people in Europe were diagnosed with IBD [3]. IBD was once thought to primarily affect North America, Europe, Australia, and New Zealand. However, the incidence and prevalence of IBD are increasing in newly industrialized countries within Asia, South America, and the Middle East [3].

The development of IBD is posited to be multifactorial. Several important risk factors for the development of IBD include certain genetic variants, immunologic deficiencies, an altered intestinal microbiome, and environmental exposures [1]. A wide range of environmental factors are thought to contribute to the pathogenesis of IBD, including tobacco use, drug use, diet, geography, and elevated psychosocial stress levels [1,3]. Among the psychosocial stress factors contributing to the pathogenesis of IBD, psychiatric stress, such as anxiety and depression, has been found to be a larger contributor to the development and exacerbation of IBD than non-psychiatric stress [4]. Anxiety levels have been observed to be significantly increased in the setting of symptomatic IBD [5]. On the other hand, lower stress levels have been observed to result in fewer relapses of symptomatic IBD [3,6].

Anxiety disorders have a high lifetime prevalence in the USA with about one-third of all adults being diagnosed with an anxiety disorder [7,8]. IBD patients are twice as likely to be diagnosed with a generalized anxiety disorder (GAD) as compared to the general population [9]. GAD is a prevalent type of anxiety; the lifetime prevalence of GAD is 6.2% for patients between the ages of 18 and 64 years old [8]. The prevalence of GAD has been rising since the start of the SARS-CoV-2 pandemic [10]. While the underlying pathophysiology of GAD is still an area of investigation, it is currently believed to result from abnormalities within the serotonergic and noradrenergic systems [11]. The first-line pharmacologic agents to treat GAD are selective serotonin reuptake inhibitors (SSRIs) and serotonin and norepinephrine reuptake inhibitors (SNRIs) [12].

The impact of GAD and other forms of anxiety on the health outcomes of IBD patients has been notable. IBD patients with symptomatic GAD have been noted to experience a lower quality of life [13]. Also, it has been observed that patients with IBD and psychiatric comorbidity require more outpatient non-psychiatric physician visits, inpatient hospital days, and medications to treat IBD [14]. A study that evaluated the prevalence of anxiety among patients hospitalized for IBD found the prevalence of anxiety to be 39.3% higher in the IBD population [15]. This same study assessing anxiety prevalence among IBD patients also found that comorbid anxiety had decreased mortality rates, but longer hospitalizations [15]. Despite the increased prevalence of anxiety among IBD patients and the connection to worse symptoms, lower quality of life and higher use of healthcare resources, the impact of anxiety on hospital outcomes has not been thoroughly explored. The objective of this study is to identify the outcomes of hospitalized IBD patients with comorbid GAD.

## Materials and methods

A retrospective cohort study was conducted to assess all patients at least 18 years old who were hospitalized for UC or CD during the year 2014. Institutional review board approval did not apply to this study given the absence of patient-level data. For this study, the data were acquired from the National (Nationwide) Inpatient Sample (NIS), a database that was created for the Healthcare Cost and Utilization Project (HCUP), sponsored by the Agency for Healthcare Research and Quality [16]. The NIS database is known to be the biggest all-payer inpatient database in the USA [16]. All of the diagnoses used in this study were identified from the NIS database utilizing the International Classification of Diseases, Ninth Edition Revision, Clinical Modification (ICD-9) codes. The data for UC and CD were pooled to collectively study IBD. The IBD patients identified for the study were then divided into two groups: a group with comorbid GAD and a group without comorbid GAD. Between these two groups, demographic information and data about their hospitalizations, including length of stay, sex, race, age, and hospitalization cost were extracted and compared. The Charlson comorbidity index, which is an established tool that is utilized to adjust for confounding variables, was also compared between the groups [17].

The statistical analyses for this study were performed utilizing the Statistical Package for the Social Sciences (SPSS) version 28.0.0 (IBM Corporation, Armonk, NY). The outcomes assessed in this study were an acute respiratory failure, sepsis, hypotension/shock, acute hepatic failure, acute renal failure (AKI), myocardial infarction (MI), acute deep vein thrombosis (DVT), ileus, inpatient mortality, colectomy, intestinal abscess, intestinal obstruction, intestinal perforation, and megacolon. A Kolmogorov-Smirnov test was used to evaluate the normal distribution of the data. For the demographic data and outcomes of the groups, independent T-tests and chi-squared tests were employed to compare the means and proportions, respectively. The statistical analyses were two-tailed with a p-value threshold of under 0.05 deemed statistically significant. Continuous variables were reported as mean ± standard deviation (SD), while categorical variables were reported as percentages (%) and numbers (N). A multivariate logistic regression analysis was also performed to elucidate if GAD is a risk factor for the aforementioned clinical outcomes, after adjusting for Charlson comorbidity index, sex, age, and race. The familywise error rate for the statistical analyses was not adjusted for given the unclear benefit of performing such a correction as well as the increased risk of type 2 statistical error associated with the correction [18].

## Results

During the year 2014, there were 24,773 adult patients hospitalized for IBD. GAD was found to be a comorbid diagnosis in 3,400 of these IBD patients. A Kolmogorov-Smirnov test showed all of the demographic data and clinical outcomes were normally distributed. As displayed in Table 1, the GAD group was younger (54.8 years old vs. 55.9 years old, p < 0.001), more likely to be female (68.6% vs. 46.3%, p < 0.001), more likely to be Caucasian (86.1% vs. 76.7%, p < 0.001), more likely to have lower total hospital expenses ($56,313 vs. $68,784, p < 0.001), and more likely to have a lower Charlson comorbidity index (2.45 vs. 2.65, p < 0.001). No significant difference in the length of stay (6.6 days vs. 6.8 days, p = 0.264) was appreciated.

**Table 1:** Demographics, characteristics, length of stay, total hospital charge, and Charles comorbidity index among inflammatory bowel disease patients with and without a history of comorbid generalized anxiety disorder.

Variable	With generalized anxiety disorder	Without generalized anxiety disorder	p-value
N = 28,173	N = 3,400	N = 24,773	
Patient age, mean (SD)	54.8 (19.2)	55.9 (21.5)	<0.001
Sex, N (%)			<0.001
Female	2,333 (68.6%)	11,478 (46.3%)	
Male	1,068 (31.4%)	13,293 (53.7%)	
Race, N (%)			<0.001
White	2,742 (86.1%)	17,782 (76.7%)	
Black	177 (5.6%)	2,307 (10.0%)	
Hispanic	18 (0.6%)	1,898 (8.2%)	
Asian or Pacific Islander	14 (0.4%)	406 (1.8%)	
Native American	73 (2.3%)	112 (0.5%)	
Other	73 (2.3%)	679 (2.9%)	
Length of stay, in days (SD)	6.6 (8.0)	6.8 (10.5)	0.264
Total hospital charges, in $ (SD)	56,313 (94,612)	68,784 (145,836)	<0.001
Charlson comorbidity index (SD)	2.45 (2.44)	2.65 (2.49)	<0.001

As seen in Table 2, unadjusted outcomes were compared between the IBD patients with and without comorbid GAD. IBD patients with comorbid GAD had a decreased risk of sepsis (9.9% vs. 13.0%, p < 0.001), acute hepatic failure (0.8% vs. 1.4%, p = 0.002), acute respiratory failure (4.4% vs. 6.0%, p < 0.001), AKI (12.9% vs. 15.7%, p < 0.001), MI (1.5% vs. 2.0%, p = 0.046), inpatient mortality (3.1% vs. 5.9%, p < 0.001), intestinal abscess (0.3% vs. 0.7%, p = 0.010), and intestinal perforation (1.5% vs. 2.3%, p = 0.007). There were no significant differences appreciated in the frequency of hypotension/shock (p = 0.967), acute DVT (p = 0.522), ileus (p = 0.077), colectomy (p = 0.828), intestinal obstruction (p = 0.084), or megacolon (p = 0.612). Due to the small sample size of patients who had megacolon in the GAD group, further analysis of this outcome was not performed.

**Table 2:** Unadjusted clinical outcomes among inflammatory bowel disease patients with and without a history of comorbid generalized anxiety disorder. *Exact number is not included in the table due to database guidelines not allowing for the reporting of a sample size of fewer than 10 patients.

Outcomes	With generalized anxiety disorder	Without generalized anxiety disorder	p-value
Hypotension/shock	401 (11.8%)	2,927 (11.8%)	0.967
Sepsis	335 (9.9%)	3,127 (13.0%)	<0.001
Acute hepatic failure	26 (0.8%)	350 (1.4%)	0.002
Acute respiratory failure	148 (4.4%)	1,479 (6.0%)	<0.001
Acute renal failure	440 (12.9%)	3,889 (15.7%)	<0.001
Myocardial infarction	50 (1.5%)	488 (2.0%)	0.046
Acute deep vein thrombosis	49 (1.4%)	393 (1.6%)	0.522
Ileus	162 (4.8%)	1,361 (5.5%)	0.077
Inpatient mortality	104 (3.1%)	1,452 (5.9%)	<0.001
Colectomy	28 (0.8%)	213 (0.9%)	0.828
Intestinal abscess	10 (0.3%)	164 (0.7%)	0.010
Intestinal obstruction	82 (2.4%)	728 (2.9%)	0.084
Intestinal perforation	52 (1.5%)	558 (2.3%)	0.007
Megacolon	*	38 (0.2%)	0.612

To further characterize the impact of GAD on the outcomes, adjusted odds ratios (aORs), which adjust for Charlson comorbidity index, sex, race, and age, were calculated. The aORs for the clinical outcomes are displayed in Table 3. Notably, GAD was found to be a risk factor for sepsis (aOR 1.33, 95% confidence interval (CI): 1.17-1.50, p < 0.001), acute hepatic failure (aOR 1.80, 95% CI: 1.18-2.73, p = 0.006), acute respiratory failure (aOR 1.24, 95% CI: 1.04-1.49, p = 0.018), inpatient mortality (aOR 1.87, 95% CI: 1.50-2.31, p < 0.001), intestinal abscess (aOR 2.35, 95% CI: 1.20-4.61, p = 0.013), and intestinal perforation (aOR 1.44, 95% CI: 1.06-1.95, p = 0.019). The p-values for the aORs of hypotension/shock (aOR 0.94, 95% CI: 0.84-1.06, p = 0.306), AKI (aOR 1.11, 95% CI: 0.99-1.24, p = 0.083), MI (aOR 1.18, 95% CI: 0.87-1.62, p = 0.278), acute DVT (aOR 0.99, 95% CI: 0.73-1.35, p = 0.972), ileus (aOR 1.05, 95% CI: 0.88-1.24, p = 0.613), colectomy (aOR 1.06, 95% CI: 0.69-1.63, p = 0.760), and intestinal obstruction (aOR 1.20, 95% CI: 0.95-1.53, p = 0.129) were not found to be statistically significant.

**Table 3:** Multivariate logistic regression analysis of the outcomes of inflammatory bowel disease patients. *Adjusted for age, sex, race, and Charlson comorbidity index.

Outcomes	Adjusted odds ratio*	95% Confidence interval	p-value
Hypotension/shock	0.94	0.84-1.06	0.306
Sepsis	1.33	1.17-1.50	<0.001
Acute hepatic failure	1.80	1.18-2.73	0.006
Acute respiratory failure	1.24	1.04-1.49	0.018
Acute renal failure	1.11	0.99-1.24	0.083
Myocardial infarction	1.18	0.87-1.62	0.278
Acute deep vein thrombosis	0.99	0.73-1.35	0.972
Ileus	1.05	0.88-1.24	0.613
Inpatient mortality	1.87	1.50-2.31	<0.001
Colectomy	1.06	0.69-1.63	0.760
Intestinal abscess	2.35	1.20-4.61	0.013
Intestinal obstruction	1.20	0.95-1.53	0.129
Intestinal perforation	1.44	1.06-1.95	0.019

Even though Table 2 and Table 3 contain the same clinical outcomes, the data between these tables may initially appear to be in conflict. As an example, in Table 2, inpatient mortality occurred less frequently in the GAD group. In contrast, Table 3 notes that this same outcome occurred more often in patients with GAD. This difference in results between Table 2 and Table 3 is due to the data in Table 3 adjusting for numerous potential confounding factors.

## Discussion

Prior studies revealed those with IBD and comorbid anxiety have more severe IBD presentations, lower quality of life, and increased length of hospital stay [15,19]. This study aimed to elucidate the outcomes of hospitalized IBD patients with comorbid GAD. The results demonstrated that IBD patients with comorbid GAD had worse outcomes, including an increased risk of sepsis, acute hepatic failure, acute respiratory failure, intestinal abscess, intestinal perforation, and inpatient mortality. All of these outcomes can be the result of an IBD exacerbation or a secondary complication from an exacerbation [20]. One possible explanation for the worse outcomes in the GAD group is suboptimal compliance with recommended IBD therapy. In a prior study, 17.6% of IBD patients with a comorbid psychiatric disease, which primarily encompassed depression, GAD, and adjustment disorders, were only partially compliant or non-compliant with recommended medical therapy [21]. Suboptimal compliance in the setting of UC has been associated with more frequent relapses [22].

Another possible etiology for the worse outcomes in IBD patients with GAD may be a result of the inflammatory state anxiety disorders can induce. Prior research has demonstrated that GAD can cause higher levels of pro-inflammatory markers such as C-reactive protein (CRP), interleukin-2, and tumor necrosis factor-alpha [23]. CRP is a common marker for trending IBD activity, and elevated CRP has been identified as a risk factor for IBD relapse and more severe disease [24]. The dual inflammatory states of IBD and GAD may have an additive or synergistic effect, accounting for the outcomes of this study.

One other explanation that can be considered for the worse outcomes in the GAD group could be due to intestinal dysmotility. IBD is a risk factor for inducing dysmotility [25]. Both depression and anxiety, as well as the SSRIs used to treat these conditions, can alter intestinal motility [26,27]. Given this impact on motility, GAD and its pharmacologic agents have the potential to worsen pre-existing dysmotility in IBD patients. Intestinal dysmotility has been associated with alterations in patients’ natural gut microbiomes [28]. Disruption of the gut microbiome, characterized by changes in its composition and stability, has been observed to cause an increased potential for IBD relapses and possibly more severe disease, therefore increasing the likelihood of a negative outcome for patients [26,29].

Of interest, the inpatient mortality data of this study on initial evaluation may seem to contradict the findings of prior literature [15]. In Tarar et al., the aOR for inpatient mortality was 0.81 with a statistically significant p-value, demonstrating anxiety was protective in the setting of IBD [15]. In contrast, for this study, the mortality aOR was 1.87 indicating GAD is a risk factor for inpatient mortality in IBD patients. The finding of anxiety being protective in Tarar et al. may possibly be related to the general “anxiety” comorbidity used in that study, which can encompass different anxiety diagnoses, duration of symptoms, and severities [15]. It is possible that many of the IBD patients with “anxiety” may have had minimally severe anxiety, anxiety that was not persistent, or only short-term anxiety. On the other hand, GAD, the primary comorbidity assessed in this study, is defined by at least six months of frequent excessive worry that is difficult to control, as well as several other symptoms impacting the quality of life, such as restlessness, fatigue, poor concentration, irritability, muscle tension, and insomnia [30].

This study was affected by several limitations. The functionality of performing research using the NIS database was a noteworthy limitation. Studies performed with the NIS database are dependent on precise billing from healthcare providers, so billing errors can result in over or under-representation of IBD patients with GAD in addition to the demographics and outcomes evaluated in this study. Moreover, due to the lack of links to medical records in NIS in order to protect privacy, the validity of billing codes for NIS research cannot be confirmed. An additional limitation was the inability to control for the severity of GAD due to a lack of ICD-9 codes for different anxiety severity levels. Knowing the anxiety levels would have allowed clarification about the relationship between the severity of anxiety and the outcomes explored in this study. Lastly, this study only evaluated the results of hospitalized IBD patients. However, both GAD and IBD are disorders that encompass both inpatient and outpatient management. Therefore, the impact of GAD on outpatients with IBD, who likely have a lower disease severity, was not captured by this study. A significant strength of the study was its capability to analyze demographic data and outcomes on a nationwide scale. In addition, another strength was this study’s utilization of a multivariate logistic regression analysis, which was able to adjust for numerous potential confounding factors.

## Conclusions

In summary, this study revealed that GAD was a risk factor for sepsis, acute hepatic failure, acute respiratory failure, intestinal abscess, intestinal perforation, and inpatient mortality in hospitalized IBD patients. Given that IBD patients with comorbid GAD are at risk for experiencing numerous worse outcomes, including increased mortality, a heightened awareness for early signs of possible complications is necessary. In the same spirit of caution, having a lower threshold to escalate the level of care for IBD patients with GAD should be considered to monitor for the development of serious complications. The results of this study could become increasingly pertinent as the prevalence of both IBD and GAD continues to increase. Additional research is needed to clarify the impact of anxiety severity on the outcomes in IBD patients with GAD and the role of anxiolytics to reduce the risks of these outcomes.
